# Diarrhea as a Presenting Symptom of Disseminated Toxoplasmosis

**DOI:** 10.1155/2017/3491087

**Published:** 2017-06-20

**Authors:** Matthew Glover, Zhouwen Tang, Robert Sealock, Shilpa Jain

**Affiliations:** Baylor College of Medicine, Houston, TX, USA

## Abstract

Disseminated toxoplasmosis is uncommon in both immunocompetent and immunocompromised hosts with gastrointestinal involvement being rarely described. We report a case of disseminated gastrointestinal toxoplasmosis in an immunocompromised man who presented with one month of diarrhea and abdominal pain. Imaging showed thickening of the ascending colon and cecum. Esophagogastroduodenoscopy and colonoscopy biopsies revealed* Toxoplasma gondii*, confirmed by immunostain. Symptoms completely resolved following treatment with pyrimethamine, sulfadiazine, and leucovorin. This case highlights the importance of including toxoplasmosis in the differential diagnosis of any immunocompromised individual presenting with gastrointestinal symptoms.

## 1. Introduction

Gastrointestinal toxoplasmosis is a rare manifestation of a relatively common disease. Disseminated* Toxoplasma gondii* must be considered in the differential diagnosis of any immunocompromised individual presenting with nonspecific gastrointestinal symptoms, particularly if from or traveling from a region with high* T. gondii* seropositivity. A biopsy is necessary for definitive diagnosis.

## 2. Case Report

**Figure 1:**
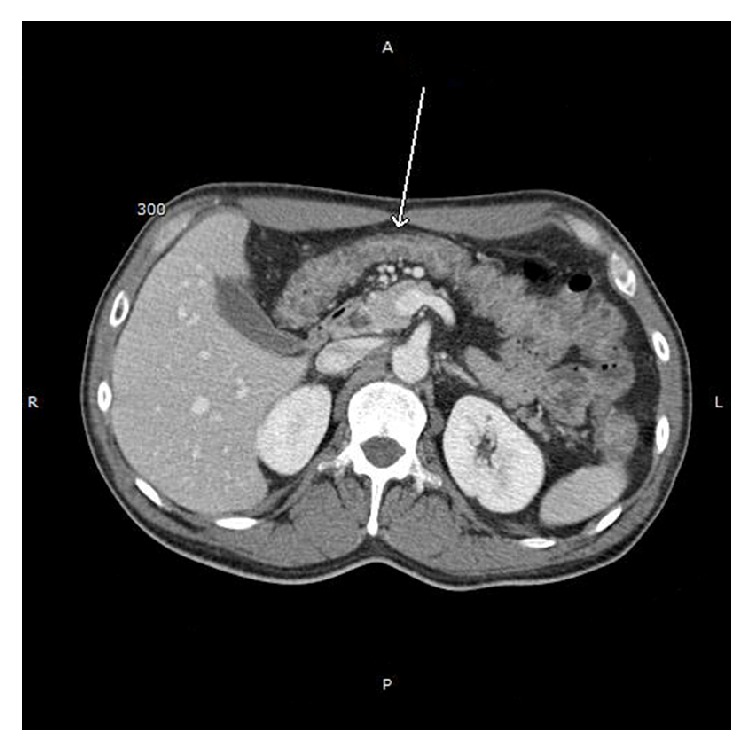
Abdominal computed tomography showing diffuse circumferential thickening of the colonic wall. The arrows refer to circumferential thickening of the transverse colon.

**Figure 2:**
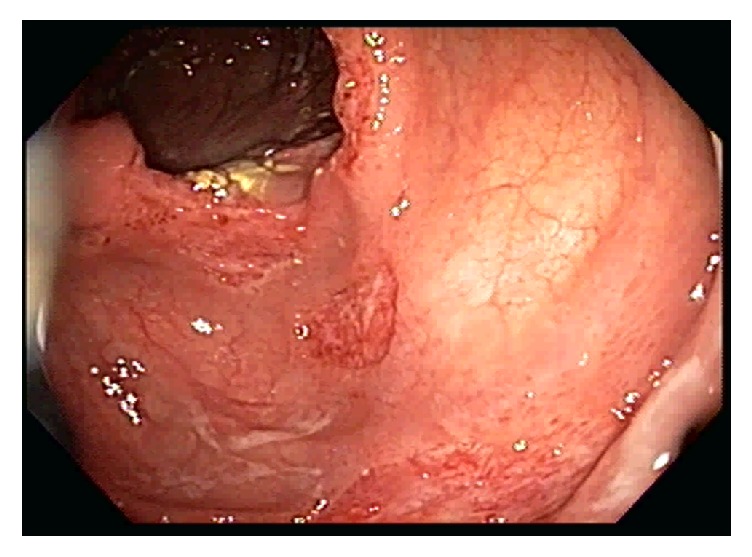
Colonoscopy with ulcerations in the cecum and ascending colon.

**Figure 3:**
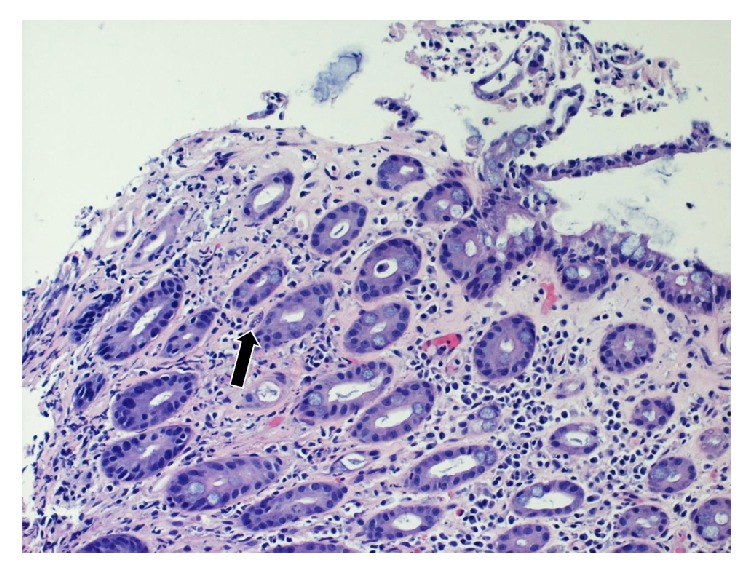
Colonic ulcer biopsy demonstrating ischemic morphology with hyalinization of the lamina propria and atrophic crypts. Small cystic forms are evident. The arrows refer to toxoplasmosis cyst present within the colonic ulcer biopsy.

**Figure 4:**
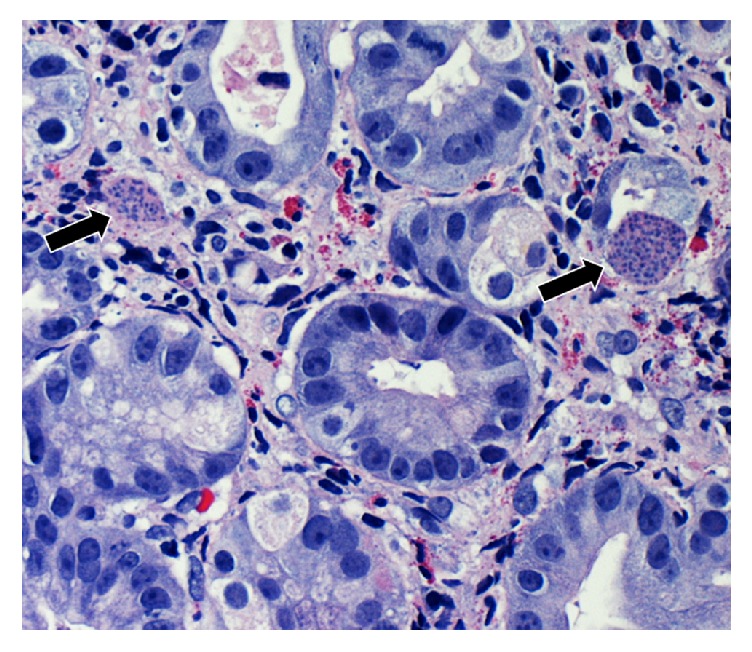
Gastric biopsy specimen showing large cystic forms present in the glandular epithelium and stroma within a background of eosinophils. The arrows refer to toxoplasmosis cyst present within the gastric biopsy.

**Figure 5:**
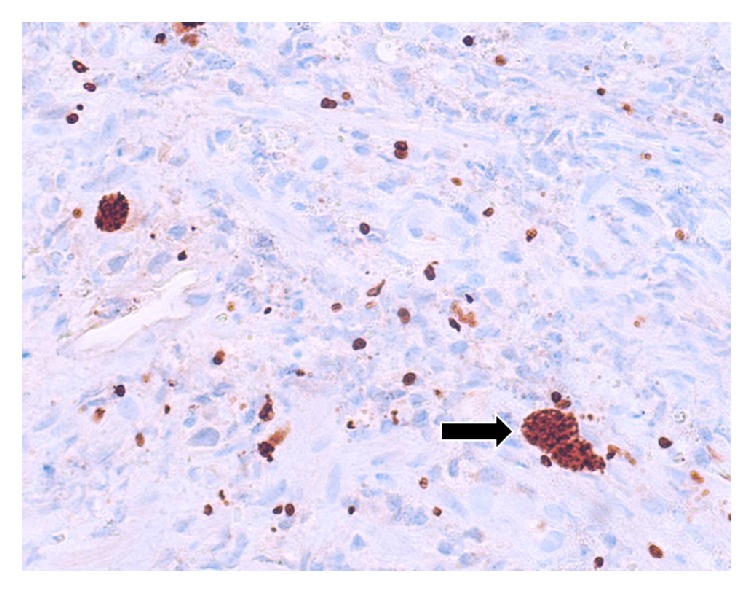
Pathologic specimen with confirmation of* T. gondii* by immunohistochemistry. Cystic forms are present alongside dispersed tachyzoites. The arrows refer to toxoplasmosis cyst highlighted by immunohistochemistry.

A 54-year-old man who recently immigrated from El-Salvador with a past medical history of Human Immunodeficiency Virus (HIV) presented with one-month progressive diarrhea, abdominal pain, subjective fevers, and 30-pound weight loss. There were no neurologic complaints. He was immunocompromised with an absolute CD4 count of 6 cells/*μ*L after being off antiretroviral therapy for the past 10 years. He had been working full time as a car mechanic and reported having multiple indoor pet cats throughout his life. The patient was cachectic and frail appearing but with an otherwise unremarkable physical exam. Laboratory showed a HIV viral load of >130,000 copies/mL with* Toxoplasma* IgG > 700 IU/mL. Laboratory was unremarkable otherwise with* Toxoplasma* IgM < 3 AU/mL, no fecal ova or parasites observed, negative cryptococcal antigen, negative enteric cultures, and a nonreactive rapid plasma reagin. CT of the chest, abdomen, and pelvis demonstrated diffuse circumferential colonic wall thickening with prominent mesenteric lymph nodes (Figure 1). Bidirectional endoscopy was performed with esophagogastroduodenoscopy showing large ulcerations in the gastric cardia and fundus. Severe inflammation and ulcerations in the right colon and cecum were noted on colonoscopy (Figure 2). Biopsies were taken from both the gastric and colonic ulcerations. Histologic examination revealed markedly active gastritis and colitis with ulceration and ischemic changes (Figure 3). Many free tachyzoites and encysted forms were identified in the lamina propria as well as the cytoplasm of epithelial, endothelial, and smooth muscle cells (Figures 3, 4, and 5). Immunohistochemistry (polyclonal rabbit anti-*Toxoplasma*, 1 : 200; Dako Corporation, Carpinteria, Calif) confirmed the diagnosis of toxoplasmosis (Figure 5). Follow-up CT head demonstrated a 1.6 cm peripherally enhancing lesion consistent with toxoplasmosis. There was subtle surrounding vasogenic edema without midline shift. The patient was diagnosed with disseminated toxoplasmosis and treatment was initiated with pyrimethamine, sulfadiazine, and leucovorin with subsequent resolution of his gastrointestinal and intracranial toxoplasmosis.

## 3. Discussion

*T. gondii* is an obligate intracellular parasite with felines serving as the definitive host.* T. gondii* can take several forms including oocysts containing sporozoites, tachyzoites, and bradyzoites contained within tissue cysts. Oral ingestion is the primary route of adult infection through consumption of either raw meat containing cysts or water contaminated with the oocysts of infected cat feces [1]. Primary infection is asymptomatic in the vast majority of immunocompetent patients [2]. Infection becomes latent in the form of tissue cysts and reactivation is rare. In immunocompromised individuals however, reactivation of latent infection is the most common cause of symptomatic toxoplasmosis. Patients with Acquired Immunodeficiency Syndrome (AIDS) and a CD4 count < 100 cells/*μ*L who are* T. gondii* seropositive without prophylaxis have a 30% chance of developing reactivated toxoplasmosis [3]. The most common site of reactivation is the central nervous system followed by the eye, myocardium, skeletal muscle, lungs, bone marrow, and peripheral blood [2, 4]. Extracranial toxoplasmosis occurs in less than 2% of all immunocompromised individuals with reactivation [5, 6].

Although the gastrointestinal tract serves as the most common port of entry into the adult human, gastrointestinal manifestations of toxoplasmosis are rare, occurring in only 6–20% of patients with disseminated disease (defined as affecting >1 organ system) [1, 4, 7–9]. The reason for this paradoxical sparing remains unclear. Antemortem diagnosis is rare with only a handful of cases ever described [10]. When gastrointestinal toxoplasmosis has been identified, the most common underlying risk factor has been severe immunosuppression, with a CD4 count < 60 cells/*μ*L [4, 5, 11]. Previous studies have found between 6.5% and 9.7% of all AIDS patients to have evidence of gastrointestinal toxoplasmosis at time of autopsy [9, 12]. Disseminated toxoplasmosis is often difficult to diagnose due to its nonspecific symptoms and requisite immunohistochemical techniques. For this reason, gastrointestinal toxoplasmosis is often identified postmortem leading to a likely underestimation of its prevalence [13]. Symptoms of gastrointestinal toxoplasmosis include diarrhea, abdominal pain, nausea, vomiting, anorexia, and ascites [14]. Complete or partial involvement of the gastrointestinal tract may be present [15]. If toxoplasmosis is suspected, biopsies are necessary as disseminated toxoplasmosis is almost invariably fatal if left untreated [11, 16]. The most common endoscopic findings of gastrointestinal toxoplasmosis are thickened gastric folds, ulcerative lesions, and nonspecific inflammation [4, 14, 17, 18].

Primary infection with* T. gondii* initially gives an IgM immune response which is followed by a* T. gondii* specific IgG response. These IgG antibodies last for life, resulting in lifelong seropositivity [19]. Seropositivity rates vary with geography and age with older individuals and those living in tropical climates more likely to be seropositive [1, 20]. With particular relevance to this case report, one study in El-Salvador found a 97% seropositivity rate by the 6th decade of life with an overall population seropositivity of 59% [21]. This is in stark contrast with a 12.4% overall seropositivity rate in the United States [22]. Although it remains impossible to say whether this particular case was primary versus reactivated toxoplasmosis, given the patients age, immigration history, and immunocompromised state, it is likely he was* T. gondii* seropositive at time of immigration arguing for reactivation over primary infection.

Unfortunately, data on treatment of extracerebral toxoplasmosis remains lacking. At current, disseminated treatment consists of the standard regimens typically used for cerebral toxoplasmosis. In all patients with diagnosed extracranial toxoplasmosis, even in the absence of neurologic symptoms, occult intracranial toxoplasmosis must be ruled out as up to 41% may have central nervous system involvement [5].

In summary, gastrointestinal toxoplasmosis is a rare manifestation of a relatively common disease. As demonstrated by this case report, disseminated* T. gondii* must be considered in the differential diagnosis of any immunocompromised individual presenting with nonspecific gastrointestinal symptoms, particularly if from or traveling from a region with high* T. gondii* seropositivity. A biopsy is necessary for definitive diagnosis.
